# Foam-free production of Surfactin via anaerobic fermentation of *Bacillus subtilis* DSM 10^T^

**DOI:** 10.1186/s13568-015-0107-6

**Published:** 2015-03-17

**Authors:** Judit Willenbacher, Jens-Tilman Rau, Jonas Rogalla, Christoph Syldatk, Rudolf Hausmann

**Affiliations:** Institute of Process Engineering in Life Sciences, Section II: Technical Biology, Karlsruhe Institute of Technology (KIT), Engler-Bunte-Ring 1, 76131, Karlsruhe, Germany; Institute of Food Science and Biotechnology (150), Section Bioprocess Engineering (150 k), University of Hohenheim, Garbenstr. 25, 70599 Stuttgart, Germany

**Keywords:** Surfactin, Anaerobic fermentation, *Bacillus subtilis*, Foam-free

## Abstract

Surfactin is one of the most popular biosurfactants due to its numerous potential applications. The usually aerobic production via fermentation of *Bacillus subtilis* is accompanied by vigorous foaming which leads to complex constructions and great expense. Therefore it is reasonable to search for alternative foam-free production processes. The current study introduces a novel approach to produce Surfactin in a foam-free process applying a strictly anaerobic bioreactor cultivation. The process was performed several times with different glucose concentrations in mineral salt medium. The fermentations were analyzed regarding specific (q_Surfactin_, vol. q_Surfactin_) and overall product yields (Y_P/X_, Y_P/S_) as well as substrate utilization (Y_X/S_). Fermentations in which 2.5 g/L glucose were employed proofed to be the most effective, reaching product yields of Y_P/X_ = 0.278 g/g. Most interesting, the product yields exceeded classical aerobic fermentations, in which foam fractionation was applied. Additionally, values for specific production rate q_Surfactin_ (0.005 g/(g∙h)) and product yield per consumed substrate (Y_P/S_ = 0.033 g/g) surpass results of comparable foam-free processes. The current study introduces an alternative to produce a biosurfactant that overcomes the challenges of severe foaming and need for additional constructions.

**Electronic supplementary material:**

The online version of this article (doi:10.1186/s13568-015-0107-6) contains supplementary material, which is available to authorized users.

## Introduction

Biosurfactants become increasingly attractive based on their biodegradability and production on the basis of renewable resources (Banat et al. 2010). Surfactin is one of the most popular biosurfactants and was already discovered in 1968 by Arima et al. (1968). The lipopeptide consists of a seven amino acid peptide ring comprising a β-hydroxy fatty acid. Surfactin is produced by *Bacillus subtilis*, a gram positive bacterium known for its application in several industrial processes, for instance the production of detergent enzymes and others (Schallmey et al. 2004). The molecule exhibits various different characteristics, which might lead to several applications. For instance, Surfactin was shown to improve plant self-resistance mechanism against soil bacteria (Ongena et al. 2007) or vigorously affects mycoplasma cells (Vollenbroich et al. 1997). Therefore, next to an application in detergents, washing agents or food products, a usage in agriculture or pharmaceutical products is also imaginable.

Naturally, amphiphile molecules produced by bacteria in cultivation processes accumulate at gas–liquid interfaces and lead to massive foam formation. The main challenge in cultivating microorganisms producing biosurfactants is to overcome this severe foam production. In the majority of cases foaming is handled by the addition of antifoam agents. Unfortunately, this strategy harbors several disadvantages, as antifoam agents are expensive and very hard to remove in downstream processes. The second most common method to cope with foam formation is to disrupt the foam by shear stress or pressure using foam breakers. However, this method is often insufficient and increases the overall costs for the production of biosurfactants. Another, more elegant, way to manage foaming in biosurfactant production processes is to apply foam fractionation, which was already shown by Cooper et al. 1981 (Cooper et al. 1981). This technique inverts the disadvantage into an advantage by using the accumulation of biosurfactants in the foam for *in situ* product enrichment and recovery. The Surfactin producer *Bacillus subtilis* is especially suited for the employment of foam fractionation, yielding high values in product recovery and enrichment (Willenbacher et al. 2014). Although this is a possible way to handle foam and to improve product yields, a realization in industrial scale is probably unrealistic in the near future.

Another artful approach is to avoid foaming at all instead of dealing with it. Several attempts have been made to establish foam-free fermentation processes. Ohno et al. for instance employed a solid state fermentation of recombinant *Bacillus subtilis* MI113 (pC112), using soybean curd residue as solid substrate (Ohno et al. 1995), which led to a yield of 2.0 g/kg (Surfactin per wet weight). Another attempt to produce Surfactin in a foam-free fashion implemented a membrane bioreactor (Coutte et al. 2010). A culture of *Bacillus subtilis* ATCC 21332 obtained a maximal Surfactin concentration of 0.242 g/L. However, a significant amount of Surfactin was adsorbed at the membranes and oxygen transfer was reduced significantly. In contrast, Chtioui et al. focused on a rotating disc bioreactor for the production of Surfactin, allowing *Bacillus subtilis* ATCC 21332 to grow free and immobilized in a biofilm at the same time (Chtioui et al. 2012). Aeration was realized above the fluid level, when the overgrown discs arose from the liquid. Maximal Surfactin concentrations of 0.212 g/L were obtained, but oxygen supply was limited and Fengycin concentrations surpassed Surfactin concentrations by far. While all these studies implemented innovative ideas to circumvent foaming, those processes are either difficult to scale up or lack high specificity.

*Bacillus subtilis* was for a long time believed to be a strict aerobic bacterium. Since 1995 research on the anaerobic growth behavior of *Bacillus subtilis* increased dramatically (Hoffmann et al. 1995; Nakano et al. 1997). By using nitrate as the terminal electron acceptor, *Bacillus subtilis* is able to perform anaerobic respiration via a nitrate reductase encoded by operon *narGHJI* (Ramos et al. 1995). In this manner nitrate is reduced to nitrite, which thereafter is transformed to ammonium via a nitrite reductase encoded by *nasDEF* (Nakano et al. 1998).

The production of biosurfactants under anaerobic conditions was already shown in 1985. The study presents the production of an undefined biosurfactant by *Bacillus licheniformis* in glucose mineral salt medium (Javaheri et al. 1985). The cultivation was performed in shake flasks, in the course of which the decreasing surface tension (from 70 mN/m to 28 mN/m) was measured. Although the characterization of the biosurfactant was only performed by thin layer chromatography and no high pressure liquid chromatography (HPLC) was applied, Javaheri et al. laid the foundation of anaerobic biosurfactant production. Subsequently, Davis et al. investigated the impact of nitrogen, carbon and oxygen conditions on Surfactin production of *Bacillus subtilis* ATCC 21332 (Davis et al. 1999). Interestingly, maximal product yields were obtained under nitrate-limited and oxygen-depleted conditions (Y_P/X_ = 0.075), which gives a further impulse to examine anaerobic Surfactin production. The proof of concept was provided by Zhang et al., who produced Surfactin with *Bacillus subtilis* ATCC 21332 strictly anaerobic for the first time (Zhang et al. 2007). The investigation focused on a connected shake flask system, introducing a nitrogen flow to induce vigorous foaming. The foam was channeled through several flasks with distilled water to collect the produced biosurfactant. While these studies demonstrate that anaerobic production of Surfactin is possible, none of them propose a solution to overcome foaming.

**Figure 1:**
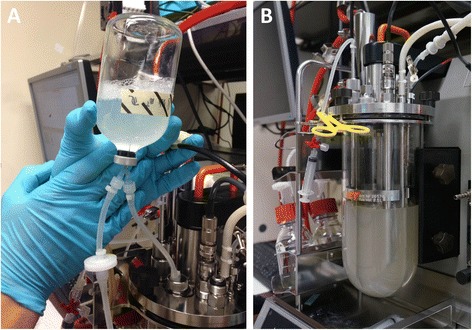
**Inoculation and fermentation of** ***Bacillus subtilis*** **DSM 10** ^**T**^ **in 2.5 L benchtop bioreactor. A**. Direct inoculation of the benchtop fermenter using a serum bottle with preculture. Nitrogen was introduced into the serum bottle via a small filter creating excess pressure inside the bottle. A second tube was used to channel the preculture directly into the inoculum device. **B**. Foam-free cultivation of *Bacillus subtilis* DSM 10^T^ applying an anaerobic fermentation process.

The aim of the current study is to combine the relatively new research field of anaerobic biosurfactant production with a foam-free bioprocess strategy (Figure 1 B). Therefore the anaerobic growth behavior of *Bacillus subtilis* DSM 10^T^ was investigated in a 2.5 L benchtop bioreactor without any gas flow through the liquid phase. Four different glucose concentrations were tested and evaluated regarding their influence on Surfactin production. The processes were analyzed focusing on maximal Surfactin concentrations (c_Surfactin_), growth rates (μ_max_), product and substrate yields (Y_P/X,_ Y_X/S,_ Y_P/S_), specific production rates (q_Surfactin_) and specific volumetric production rates (vol. q_Surfactin_).

## Materials and methods

### Chemicals

All chemicals applied in the current study were of analytical grade and purchased from Carl Roth GmbH (Karlsruhe, Germany). The Surfactin standards for HPLC analysis were obtained from Sigma-Aldrich Laborchemikalien GmbH (Seelze, Germany).

### Microorganism and strain maintenance

The wildtype strain *Bacillus subtilis* DSM 10^T^ was used for all experiments during this study. The microorganism was obtained from the DSMZ (Deutsche Sammlung von Mikroorganismen und Zellkulturen GmbH, Braunschweig, Germany) and stored as glycerol stocks, prepared from a culture in Lysogeny Broth (Bertani 1951) from the exponential growth phase, at −80°C.

### Culture conditions

#### Media

The employed mineral salt medium was based on the fermentation medium of Cooper (Cooper et al. 1981): 8.0 × 10^−4^ M MgSO_4_, 7.0 × 10^−6^ M CaCl_2_, 4.0 × 10^−6^ M FeSO_4_, 4.0 × 10^−6^ M Na_2_EDTA, 1 × 10^−6^ M MnSO_4_. In contrast to the original medium (40 g/L glucose) the concentration of glucose was altered to 2.5 g/L, 5 g/L, 7.5 g/L and 10 g/L, during various cultivations. Furthermore, the former nitrogen source 0.05 M NH_4_NO_3_ was replaced with 0.1 M NH_4_Cl and 0.1177 M NaNO_3_. The deployed concentration of the phosphate buffer demanded slight changes depending on its usage for inoculum cultures or fermentation processes. For the cultivation in serum bottles the original 0.07 M phosphate buffer (0.03 M KH_2_PO_4_ and 0.04 M Na_2_HPO_4_) was used, whereas for the cultivation in benchtop bioreactors a 0.01 M phosphate buffer was employed (4.29 × 10^−3^ M KH_2_PO_4_ and 5.71 × 10^−3^ M Na_2_HPO_4_).

The preparation of medium for the cultivation in serum bottles demanded a different approach compared to the preparation of medium for the cultivation in benchtop bioreactors. Four different stock solutions were prepared for the cultivation in serum bottles. One stock solution contained the salt compounds (NH_4_Cl, NaNO_3_, KH_2_PO_4_, Na_2_HPO_4_) and was later completed to the final volume of 50 or 100 mL, respectively. The second stock solution included a 5.56-fold glucose solution of the final glucose concentration. In comparison, the third and fourth stock solution contained a 50-fold MgSO_4_ solution and a 1000-fold solution of the trace elements (CaCl_2_, FeSO_4_, Na_2_EDTA, MnSO_4_). All solutions were filled into separate serum bottles and anaerobic conditions were adjusted by 20 alternating cycles of purging with gas (20 vol.-% CO_2_ in N_2_, 45 s) and evacuating (70 mbar, 45 s). Subsequently the bottles were autoclaved and the salt stock solution was completed under anaerobic conditions to receive the final concentrations of glucose, MgSO_4_ and trace elements.

**Figure 2:**
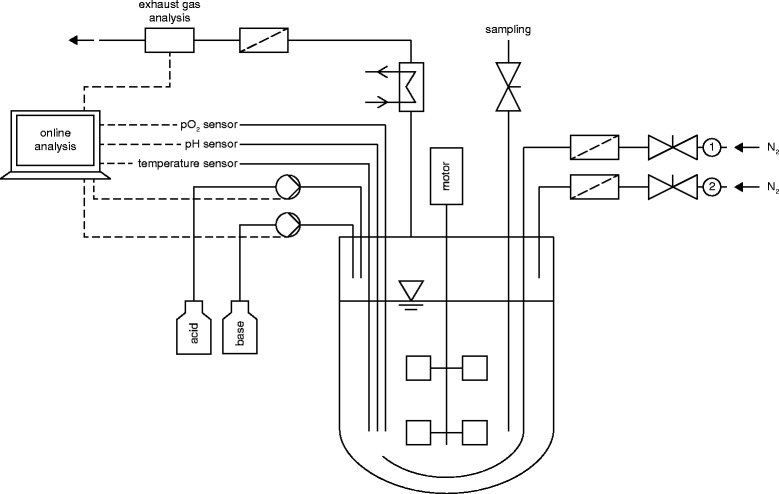
**Model of the employed fermentation system.** A 2.5 L benchtop bioreactor was used for anaerobic cultivation of *Bacillus subtilis* DSM 10^T^. The bioreactor was equipped with two Rushton turbines, a temperature sensor, pH and pO_2_ electrodes, peristaltic pumps for pH control, an exhaust cooler and attached exhaust gas analysis, which were connected to a computer for online analysis. To adjust anaerobic conditions in the liquid medium and the head space of the bioreactor valve 1 was opened to allow a N_2_ flow through the sparger. During fermentation valve 1 was closed and N_2_ was allowed to flow through valve 2, enabling a constant gas flow through the head space.

For the preparation of the bioreactor medium four stock solutions were prepared in a similar fashion. However, the first stock solution (NH_4_Cl, NaNO_3_, KH_2_PO_4_, Na_2_HPO_4_) was autoclaved inside the bioreactor. Whereas the glucose, MgSO_4_ and trace elements stock solutions were prepared and autoclaved in separate vessels. The medium was completed inside the bioreactor after sterilization and thereafter anaerobic conditions were reached by purging the bioreactor with N_2_ (4 Lpm, 1050 rpm, 20 min, Figure 2: valve 1 open).

#### Preparation of inoculum cultures

For the preparation of the first seed culture a loop of *B. subtilis* DSM 10^T^ from the glycerol stock solution was inoculated in 20 mL of Lysogeny Broth (inside a 100 mL baffled shake flask) and incubated in a shake incubator chamber (Multitron II, HT Infors, Bottmingen, Switzerland) at 30°C and 120 rpm for 24 h. The second seed culture was inoculated with a resulting OD_600_ of 0.05 under anaerobic conditions in prepared serum bottles with 50 or 100 mL of mineral salt medium, respectively. The serum bottles were incubated in a horizontal position but otherwise in the same manner as the first seed culture. After 24 h of incubation approximately 200 mL of the second seed culture were used to inoculate the aqueous phase of the bioreactor (Figure 1 A). The initial OD_600_ inside the bioreactor fluctuated between 0.03 and 0.07, depending on bacterial growth of the second seed culture.

### Cultivation in a 2.5 L benchtop bioreactor

All cultivations were carried out in 2.5 L benchtop bioreactors (Minifors, HT Infors, Bottmingen, Switzerland) with 1.0 L mineral salt medium. The bioreactors were equipped with pH (Mettler-Toledo International Inc., Greifensee, Switzerland) and pO_2_ electrodes (Oxyferm, Hamilton Bonaduz AG, Bonaduz, Switzerland), a temperature sensor and Rushton turbines. The temperature was adjusted to 30°C and the pH was controlled to a value of 7.0 by the addition of 4 M NaOH or 4 M H_3_PO_4_ (Figure 2). The stirrer was adjusted to 300 rpm the entire time of cultivation. The medium was not exposed to gas flow throughout the whole fermentation process to guarantee an absolutely foam-free cultivation. However, to avoid reflux of air through the exhaust cooler and to allow the measurement of CO_2_ through the exhaust gas analysis, a constant N_2_ gas flow through the headspace of the bioreactor with 0.1 Lpm (1.5 L headspace volume) was adjusted (Figure 2: valve 2 open).

The fermentation process was started with 1.0 L of the described mineral salt medium and the additional volume of the inoculated seed culture (200 mL). Since the bioreactor cultivation was realized as a batch cultivation, no further medium components were added. During the cultivation pH, pO_2_, CO_2_ exhaust, temperature, stirrer speed and addition of acid and base were consistently monitored (Figure 2). Samples were taken from the cultivation broth (4 mL) without allowing any air flow inside the bioreactor. All fermentations were performed as duplicates.

## Analytical methods

### Sampling and sample processing

By day samples were taken every three hours, whereas during nights the intervals were between five and seven hours. The sampling was designed to prevent air from entering the bioreactor system to guarantee anaerobic conditions inside. The offline analysis of the cultivation broth samples included the determination of the OD_600_ and the glucose, nitrate and Surfactin concentration of the supernatant. The concentration of glucose and nitrate was analyzed using a glucose assay kit (Cat. No. 10 716 251 035, R-Biopharm AG, Darmstadt, Germany) and a nitrate assay kit (1.09713.0001, Merck KGaA, Darmstadt, Germany). The concentrations were determined according to the manufacturer instructions, utilizing a spectrophotometric method (Ultrospec 2100 pro, General Electric Deutschland Holding GmbH, Frankfurt, Germany). The concentration of Surfactin was determined by analyzing the sample supernatant using HPLC (Willenbacher et al. 2014).

### Data analysis

To enable the evaluation of the fermentation processes, several values were calculated to compare the different experiments. Using the results of CDW, glucose and Surfactin mass the values of Y_X/S_ [g/g], Y_P/X_ [g/g], Y_P/S_ [g/g], μ [h^−1^], q_Surfactin_ [g/(g · h)], volumetric q_Surfactin_ [g/(L · h)], were determined.

The biomass yield Y_X/S_ was defined in an integral manner, using the maximal mass of produced CDW (m_Xmax_) and the corresponding mass of depleted glucose (m_S_; Eq. 1).

1 $$ {\boldsymbol{Y}}_{\boldsymbol{X}/\boldsymbol{S}}=\frac{\Delta {\boldsymbol{m}}_{\boldsymbol{X}\boldsymbol{max}}}{\Delta {\boldsymbol{m}}_{\boldsymbol{S}}} $$

The product yield Y_P/X_ was calculated in the same manner as Y_X/S_ using the maximal mass of produced product (m_Surfactin max_) and the corresponding CDW over the whole fermentation process (Eq. 2).

2 $$ {\boldsymbol{Y}}_{\boldsymbol{P}/\boldsymbol{X}}=\frac{\Delta {\boldsymbol{m}}_{\boldsymbol{Surfactin}\ \boldsymbol{max}}}{\Delta {\boldsymbol{m}}_{\boldsymbol{Xmax}}} $$

The product yield Y_P/S_ was calculated dividing the maximal produced mass of Surfactin by the corresponding mass of consumed glucose during the entire fermentation process (Eq. 3).

3 $$ {\boldsymbol{Y}}_{\boldsymbol{P}/\boldsymbol{S}}=\frac{\Delta {\boldsymbol{m}}_{\boldsymbol{S}\boldsymbol{urfactin}\ \boldsymbol{max}}}{\Delta {\boldsymbol{m}}_{\boldsymbol{S}}} $$

The specific growth rate μ was determined in a differential manner using Eq. 4.

4 $$ \boldsymbol{\mu} =\frac{\boldsymbol{ln}\frac{{\boldsymbol{m}}_{{\boldsymbol{X}}_2}}{{\boldsymbol{m}}_{{\boldsymbol{X}}_1}}}{{\boldsymbol{t}}_2-{\boldsymbol{t}}_1} $$

The specific productivity q_Surfactin_ was calculated in an integral manner using the maximal mass of produced Surfactin, the corresponding mass of CDW and cultivation time (Eq. 5).

5 $$ {\boldsymbol{q}}_{\boldsymbol{Surfactin}}=\frac{\Delta {\boldsymbol{m}}_{\boldsymbol{Surfactin}\ \boldsymbol{max}}}{\Delta {\boldsymbol{m}}_{\boldsymbol{Xmax}}\bullet \Delta \boldsymbol{t}} $$

The integral volumetric specific productivity q_Surfactin_ was determined using the maximal mass of Surfactin, the average medium volume and cultivation time (Eq. 6).

6 $$ \boldsymbol{volumetric}\ {\boldsymbol{q}}_{\boldsymbol{Surfactin}\ \boldsymbol{max}}=\frac{\Delta {\boldsymbol{m}}_{\boldsymbol{Surfactin}\ \boldsymbol{max}}}{{\boldsymbol{V}}_{\boldsymbol{Reactor}}\bullet \Delta \boldsymbol{t}} $$

## Results

### Anaerobic growth

**Figure 3:**
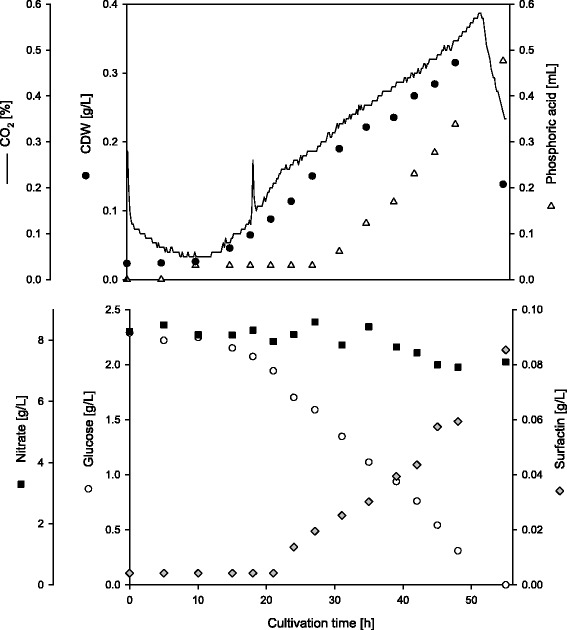
**Anaerobic fermentation of** ***Bacillus subtilis*** **DSM 10** ^**T**^ **employing 2.5 g/L glucose.** Time course of CDW [g/L], CO_2_ [%], phosphoric acid [mL], nitrate [g/L] and glucose [g/L] in comparison to produced Surfactin [g/L] during the fermentation process of *Bacillus subtilis* DSM 10^T^. The values for CDW (black circle), CO_2_ (line), phosphoric acid (grey triangle), nitrate (square), glucose (white circle) and Surfactin (grey rhombus) are given as examples of one fermentation. All graphs have been created in SigmaPlot (Systat Software Inc., San Jose, CA, USA), and are attached to this manuscript in .eps format.

Altogether eight fermentations were performed testing four different glucose concentrations as duplicates. An example is given in Figure 3, showing a fermentation of *Bacillus subtilis* DSM 10^T^ using 2.5 g/L glucose as carbon source (for exemplary fermentations using 5 g/L, 7.5 g/L and 10 g/L glucose see Additional file 1: Figure S2-S4, the fermentation employing 2.5 g/L glucose is additionally presented with matching axis scaling in Additional file 1: Figure S1). All figures present the course of the CDW, CO_2_, phosphoric acid, nitrate, glucose and Surfactin concentrations with time. The fermentation shown in Figure 3 endured 55 h. The process was terminated because the levels of CO_2_ and CDW were drastically decreasing and the glucose was completely consumed. During the fermentation the CDW continually increased reaching 0.320 g/L at its maximum. The amount of CO_2_ (no longer solved in the medium and therefore carried on within the N_2_ stream in the headspace) increased simultaneously with the CDW. Meanwhile the glucose concentration consistently decreased until its depletion. In contrast, only 1 g/L nitrate was consumed during this fermentation (during fermentations with 10 g/L glucose about 5 g/L nitrate were used up, Additional file 1: Figure S4). The concentration of Surfactin in the fermentation medium started to increase after 24 h of incubation. It reached its maximum at the end of the fermentation yielding 0.09 g/L Surfactin. The amount of added phosphoric acid to adjust the mediums pH level increased significantly after 34 h of cultivation. The demand for pH regulation is caused by *Bacillus subtilis* anaerobic metabolism. In this pathway nitrate is used as terminal electron acceptor. The reduction of nitrate to nitrite via a nitrate reductase and the additional conversion of nitrite to ammonia via a nitrite reductase results in the production of an alkaline end product. In contrast to conventional aerobic cultivations of *Bacillus subtilis*, where the addition of base marks cell growth, the addition of acid represents vivid cell growth under anaerobic conditions. The amount of dissolved oxygen was monitored throughout the fermentation processes but is not shown in the figures, because values were below detection limit.

### Comparison of process parameters during anaerobic fermentation with different glucose concentrations

The fermentations of *Bacillus subtilis* DSM 10^T^ with various glucose concentrations were analyzed regarding product yields and substrate utilization. Table 1 presents an overview of the most interesting process parameters, such as cultivation time, maximal CDW, maximal Surfactin concentration, maximal growth rate, product yields (Y_P/X_, Y_P/S_, q_Surfactin_, vol. q_Surfactin_) and substrate utilization (Y_X/S_). All illustrated values are mean values of two fermentations. The duration of the fermentation depended on the starting glucose concentration. Fermentations with 2.5 g/L glucose lasted for 55 h, whereas fermentations with 10 g/L glucose averagely endured 161 h. Fermentations with 5 g/L and 7.5 g/L glucose ran for approximately 100 h. The maximal CDW was reached during fermentations with 7.5 g/L glucose (0.856 g/L). In contrast, only 0.320 g/L CDW were yielded in fermentations with 2.5 g/L glucose. Fermentations with 5 g/L glucose or more reached at least 0.105 g/L Surfactin as maximal concentration. Fermentations with 2.5 g/L glucose earned 0.087 g/L Surfactin. The highest maximal growth rate μ_max_ was reached by fermentations with 7.5 g/L glucose (0.118 h^−1^), whereas fermentations with 10 g/L glucose only reached maximal growth rates of 0.074 h^−1^. The values of overall Y_P/X_ differed widely between the fermentations with different glucose concentrations. Fermentations with 5 g/L or 7.5 g/L glucose earned product yields around 0.17 g/g. In contrast, fermentations with 2.5 g/L and 10 g/L reached Y_P/X_ values of 0.278 g/g and 0.259 g/g, respectively. Overall values of Y_X/S_ varied around 0.1 g/g except for fermentations with 10 g/L glucose. These cultivations led to Y_X/S_ values of 0.049 g/g. The results for Y_P/S_ show much higher values for fermentations with low glucose concentrations. Fermentations with 2.5 g/L glucose reached 0.033 g/g instead of 0.011 g/g with 10 g/L glucose in mineral salt medium. Additionally, cultivations using 2.5 g/L glucose yielded high specific production rates of 0.005 g/(g∙h). Interestingly, all other fermentations reached only 0.002 g/(g∙h). Volumetric specific production rates varied for all fermentations between 0.001 g/(L∙h) and 0.002 g/(L∙h).

**Table 1:** **Summary of the process parameters during various fermentations**

**Glucose concentration [g/L]**	**2.5**	**5**	**7.5**	**10**
Cultivation time [h]	55	102	108	161
Max. CDW [g/L]	0.320	0.612	0.856	0.586
Max. c_Surfactin_ [g/L]	0.087	0.105	0.150	0.158
μ_max_ [h^−1^]	0.105	0.114	0.118	0.074
Y_P/X_ [g/g]	0.278	0.169	0.179	0.259
Y_X/S_ [g/g]	0.120	0.105	0.119	0.049
Y_P/S_ [g/g]	0.033	0.018	0.022	0.011
q_Surfactin_ [g/(g∙h)]	0.005	0.002	0.002	0.002
vol. q_Surfactin_ [g/(L∙h)]	0.002	0.001	0.002	0.001

All values are mean values of two fermentations.

Comparison of process parameters during anaerobic fermentation of *Bacillus subtilis* DSM 10^T^ with different glucose concentrations.

Although cultivations with 2.5 g/L glucose reached only small amounts of CDW and Surfactin, these fermentations are comparably efficient. The cultivation time is much shorter and values for μ_max_, Y_X/S_ and vol. q_Surfactin_ are comparatively high. Moreover, fermentations with 2.5 g/L glucose reached excellent values for Y_P/X_, Y_P/S_ and specific production rate q_Surfactin_ emphasizing an outstanding conversion of substrate into product. Nevertheless, fermentations with 2.5 g/L glucose yielded only small amounts of Surfactin, due to the short cultivation time. As a consequence it would be interesting to test whether higher overall amounts of Surfactin can be reached by applying a repeated fed-batch process. Interestingly, on closer inspections Surfactin concentrations did increase simultaneously to rising initial glucose concentrations possibly due to longer cultivation times. Surprisingly, fermentations employing 10 g/L did also achieve an almost equal value for Y_P/X_ in comparison to fermentations with 2.5 g/L glucose. But this positive result is misleading as overflow metabolism (as a result of the high initial glucose concentration) leads to low values of CDW, μ_max_ and Y_X/S_. This means that the bacterial growth is already strongly restricted under the employment of 10 g/L glucose. As a result data for Y_P/S_ and q_Surfactin_ are comparably low. These findings support the usage of lower initial glucose concentrations for the anaerobe fermentation of *B. subtilis* DSM 10^T^ for the production of Surfactin to avoid overflow metabolism.

## Discussion

### Comparison with other foam-free cultivation systems and aerobic fermentation with foam fractionation

The aim of the current study was to introduce a new approach for a foam-free biosurfactant production process. The results shown in Figure 3 and Table 1 demonstrate a high efficiency for anaerobic cultivations with low glucose concentrations. There are only three other fermentation processes described for the foam-free production of Surfactin. The solid state fermentation analyzed by Ohno et al. is incomparable with aqueous fermentations (Ohno et al. 1995), hence these data are not further discussed in comparison to the current study. However, Chtioui et al. established a rotating disc bioreactor allowing air flow only above the liquid phase. The growth of a *Bacillus subtilis* ATCC 21332 biofilm led to the production of Surfactin and Fengycin (Chtioui et al. 2012). Chtioui et al. provided several results about product yields and substrate utilization. On basis of these findings further process parameters were calculated (see Table 2) to achieve a more complete comparison with the results of the current study (Table 2). Coutte et al. introduced a novel membrane bioreactor for the production of biosurfactants (Coutte et al. 2010). The data of the *Bacillus subtilis* ATCC 21332 cultivation were also used for the calculation of additional process parameters (Table 2). Therefore, Table 2 compares the data of three different foam-free fermentation processes for the production of Surfactin. To outline the differences between these methods and a traditional aerobic cultivation for the production of Surfactin, these results are additionally collated with a fermentation process applying foam fractionation (Willenbacher et al. 2014).

**Table 2:** **Summary of the process parameters of different foam-free processes**

	**Chtioui et al. ** 2012	**Coutte et al.** 2010	**The current study**	**Willenbacher et al. ** 2014
Surfactin producer	*Bacillus subtilis* ATCC 21332	*Bacillus subtilis* ATCC 21332	*Bacillus subtilis* DSM 10^T^	*Bacillus subtilis* DSM 10^T^
Fermentation approach	Rotating discs	Membrane bioreactor	Anaerobic, no gas flow	Foam fractionation
Cultivation time [h]	72	72	55	30
Max. c_Surfactin_ [g/L]	0.212*	0.242*	0.087	3.995 (foam)
Y_P/X_ [g/g]	0.068	0.078*	0.278	0.192
Y_X/S_ [g/g]	0.189	0.164*	0.120	0.268
Y_P/S_ [g/g]	0.013	0.013	0.033	0.052
q_Surfactin_ [g/(g∙h)]	0.001*	0.001*	0.005	0.006
vol. q_Surfactin_ [g/(L∙h)]	0.003*	0.003*	0.002	0.018

*the values were calculated during the current study, using data of Chtioui et al. 2012 and Coutte et al. 2010 (m_Surfactin_, CDW, cultivation time and cultivation volume).

Comparison of different foam-free Surfactin production processes regarding their process parameters and collation with a fermentation process applying foam fractionation [2].

The processes of Chtioui et al. and Coutte et al. each yielded above 0.2 g/L Surfactin. Whereas only 0.087 g/L Surfactin were reached in the current study (with 2.5 g/L glucose in mineral salt medium). However, the fermentations of Chtioui et al. and Coutte et al. lasted comparatively longer (72 h instead of 55 h). Aerobic fermentations with *Bacillus subtilis* using foam fractionation take much shorter time (30 h) and yield much higher concentrations in foam (3.995 g/L). Values for Y_X/S_ differ only slightly between the foam-free processes (0.120 g/g – 0.189 g/g), but are relatively low compared to cultivations applying foam fractionation (0.268 g/g). The results for volumetric production rates are very similar, too, between the foam-free fermentations (0.002 g/(L∙h) – 0.003 g/(L∙h)). The foam fractionation fermentation reached a much higher value for vol. q_Surfactin_ in comparison (0.018 g/(L∙h)). The product yield in contrast to substrate utilization is given by the parameter Y_P/S_. The values for cultivations of Chtioui et al. and Coutte et al. are both 0.013 g/g. The current study reached a much higher value of 0.033 g/g for Y_P/S_. However, fermentations employing foam fractionation still yield higher Y_P/S_ values (0.052 g/g). The specific production rate q_Surfactin_ is five-times higher in anaerobic fermentations using 2.5 g/L glucose (0.005 g/(g∙h)) in comparison to other foam-free fermentations (0.001 g/(g∙h)). Aerobic processes applying foam fractionation yield rather similar results for q_Surfactin_ (0.006 g(g∙h)). Most surprising are the results for Y_P/X_. Fermentations of Chtioui et al. and Coutte et al. reached 0.068 g/g and 0.078 g/g, respectively. In contrast, anaerobic fermentations of the current study employing 2.5 g/L glucose yielded 0.278 g/g. These findings surpass even Y_P/X_ values of aerobic fermentations employing foam fractionation (0.192 g/g).

Interestingly, the results of Chtioui et al. and Coutte et al. show very similar values for efficiency, product yields and substrate utilization although completely different fermentation approaches were applied. This similarity was revealed only after calculating some additional process parameters from the original data of these publications (Table 2). While rotating disc bioreactors or membrane reactors seem very attractive alternatives to common foam fractionation processes the presented data in Table 2 expose their low yields in comparison to the results of a classic foam fractionation process. The comparison of the results of Chtioui et al. and Coutte et al. with data of the current study displays a much higher effectiveness of the anaerobic fermentation approach. Although overall less Surfactin was produced, much more Surfactin was produced per CDW. This implies that the bacterial growth is probably lower compared to the rotating discs or membrane bioreactors, but single cells produce more Surfactin under completely anaerobic conditions. These findings explain the much higher values for Y_P/X_, Y_P/S_ and q_Surfactin_. In comparison to an aerobic fermentation process with foam fractionation some process parameters are lower (e.g., vol. q_Surfactin_ and Y_X/S_), but values for Y_P/S_ and q_Surfactin_ are at the same level. Most important is the much higher value for Y_P/X_ under anaerobic conditions. This implies a much better production of Surfactin per CDW not only in comparison to other foam-free processes, but even in comparison to aerobic foam fractionation processes.

The current study demonstrates a new approach to produce Surfactin without any foam formation. Moreover, anaerobic cultivation and foam-free biosurfactant production are combined in one process for the first time. The anaerobic production of Surfactin was shown before, but never analyzed for product yields and substrate utilization. The comparison of different fermentations with various glucose concentrations displayed great efficiency for processes applying low glucose concentrations. Furthermore, the confrontation with other foam-free processes revealed a much higher effectiveness of the anaerobic fermentation process of the current study.

## Additional file

Additional file 1:
**Exemplary anaerobic fermentations employing 2.5 g/L, 5 g/L, 7.5 g/L and 10 g/l glucose.**
