# Supplementary material for: Foam-free production of Surfactin via anaerobic fermentation of Bacillus subtilis DSM 10T
Source: AMB Express. 2015 Mar 17;5:21. doi: 10.1186/s13568-015-0107-6 (PMC4385232; doi:10.1186/s13568-015-0107-6)
Supplement: Additional file 1: — Exemplary anaerobic fermentations employing 2.5 g/L, 5 g/L, 7.5 g/L and 10 g/l glucose. [file 13568_2015_107_MOESM1_ESM.pdf]

Additional file 1: Exemplary anaerobic fermentations employing 2.5 g/L, 5 g/L, 7.5 g/L and 10 g/L glucose

Belonging to the manuscript:

"Foam-free production of Surfactin via anaerobic fermentation of Bacillus subtilis *DSM 10*^{T}*"*

submitted to AMB Express on 2015-01-28

Judit Willenbacher[a,], Jens-Tilman Rau[a], Jonas Rogalla[a], Christoph Syldatk[a] and Rudolf Hausmann[b]*

[a]Institute of Process Engineering in Life Sciences, Section II: Technical Biology, Karlsruhe Institute of Technology (KIT), Engler-Bunte-Ring 1, 76131 Karlsruhe, Germany

[b]Institute of Food Science and Biotechnology (150), Section Bioprocess Engineering (150k), University of Hohenheim, Garbenstr. 25, 70599 Stuttgart, Germany

*****Corresponding author:	Judit Willenbacher
Address:	Engler-Bunte-Ring 1, 76131 Karlsruhe, Germany
Phone:	+49-721-608-46737
Fax:	+49-721-608-44881
Email:	judit.willenbacher@kit.edu

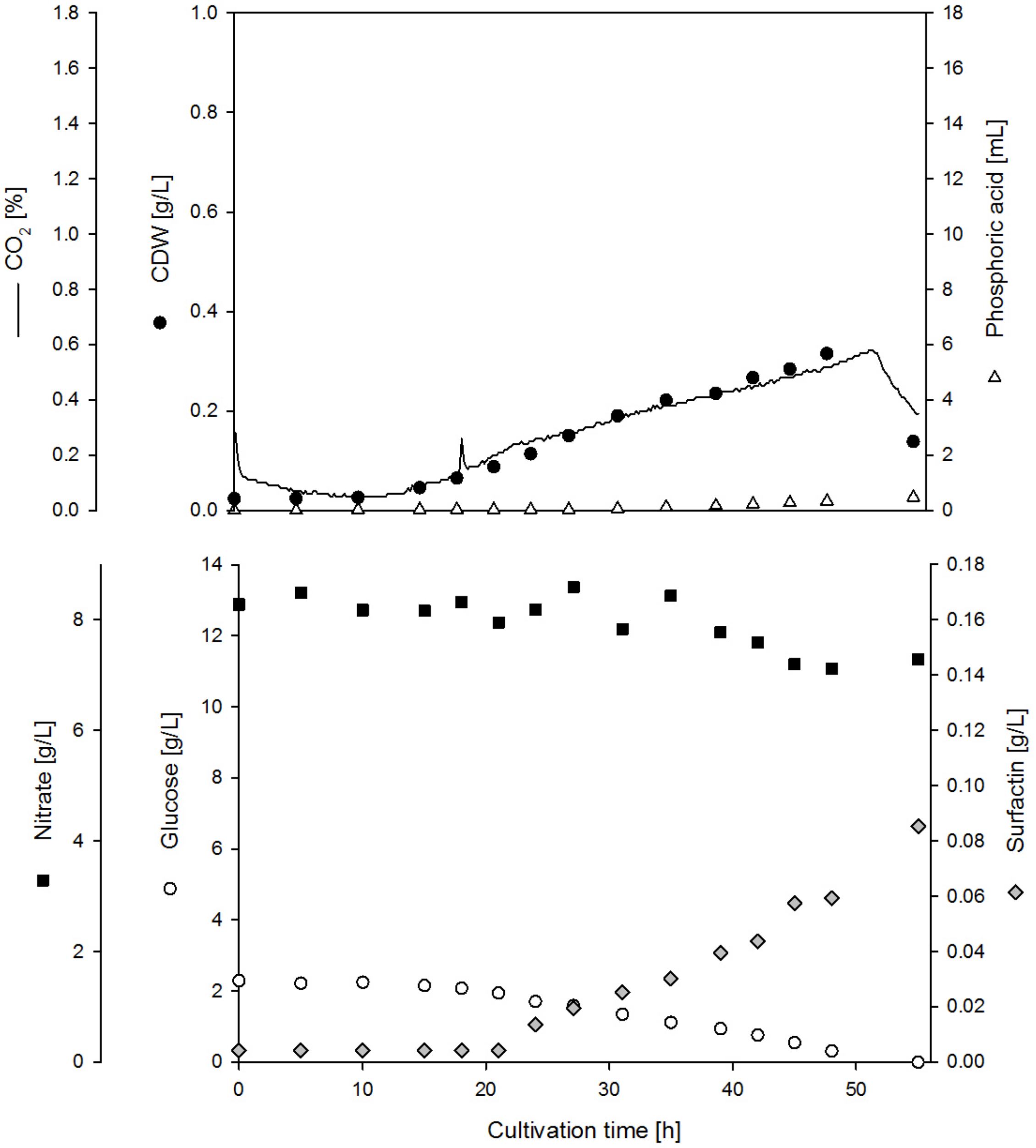

Figure S1 Anaerobic fermentation of *Bacillus subtilis* DSM 10^{T} employing 2.5 g/L glucose Time course of CDW [g/L], CO_2 [%], phosphoric acid [mL], nitrate [g/L] and glucose [g/L] in comparison to produced Surfactin [g/L] during the fermentation process of *Bacillus subtilis* DSM 10^{T} with 2.5 g/L glucose. The values for CDW (black circle), CO_2 (line), phosphoric acid (grey triangle), nitrate (square), glucose (white circle) and Surfactin (grey rhombus) are given as examples of one fermentation

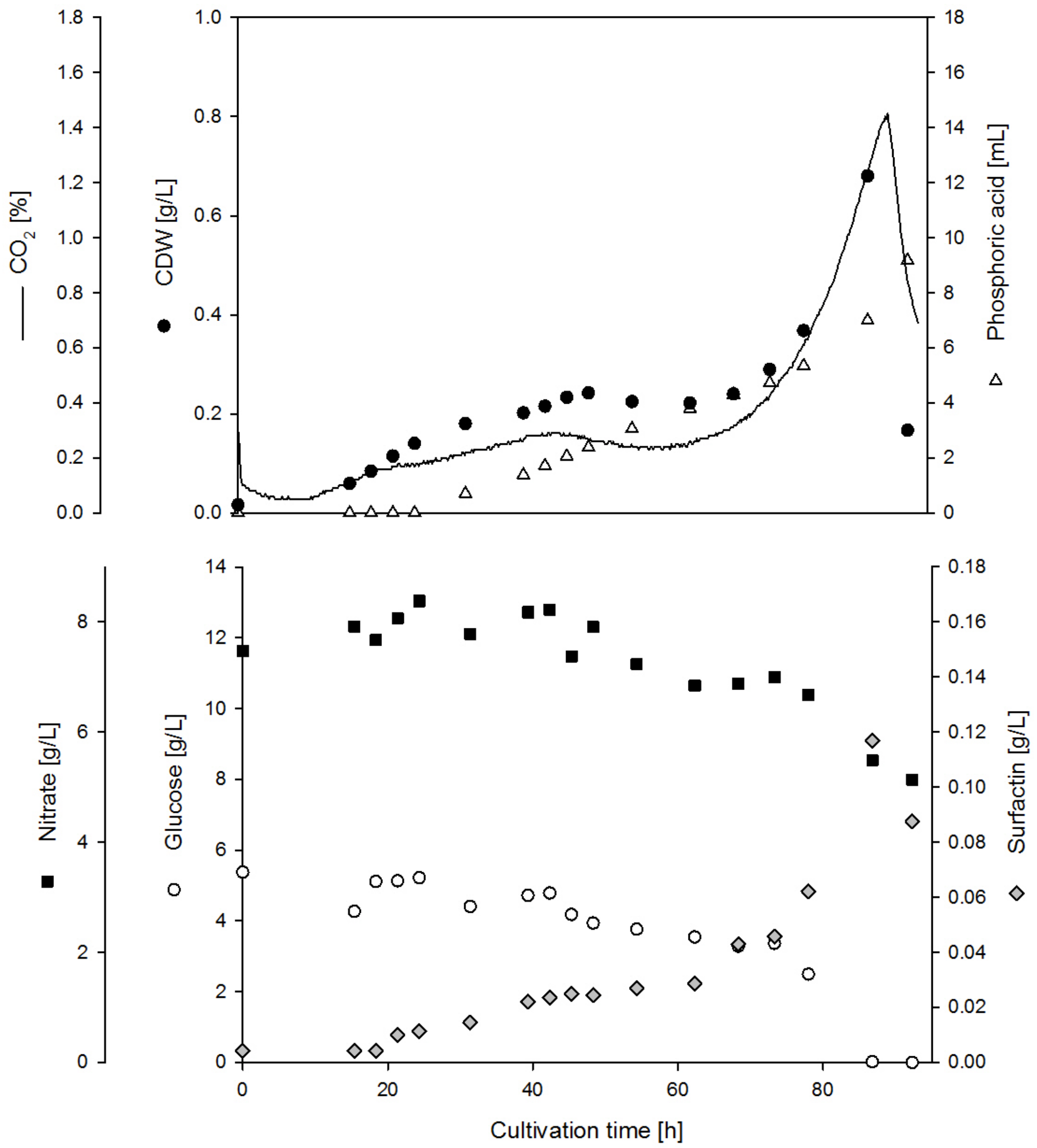

Figure S2 Anaerobic fermentation of *Bacillus subtilis* DSM 10^T employing 5.0 g/L glucose Time course of CDW [g/L], CO_2 [%], phosphoric acid [mL], nitrate [g/L] and glucose [g/L] in comparison to produced Surfactin [g/L] during the fermentation process of *Bacillus subtilis* DSM 10^T with 5.0 g/L glucose. The values for CDW (black circle), CO_2 (line), phosphoric acid (grey triangle), nitrate (square), glucose (white circle) and Surfactin (grey rhombus) are given as examples of one fermentation

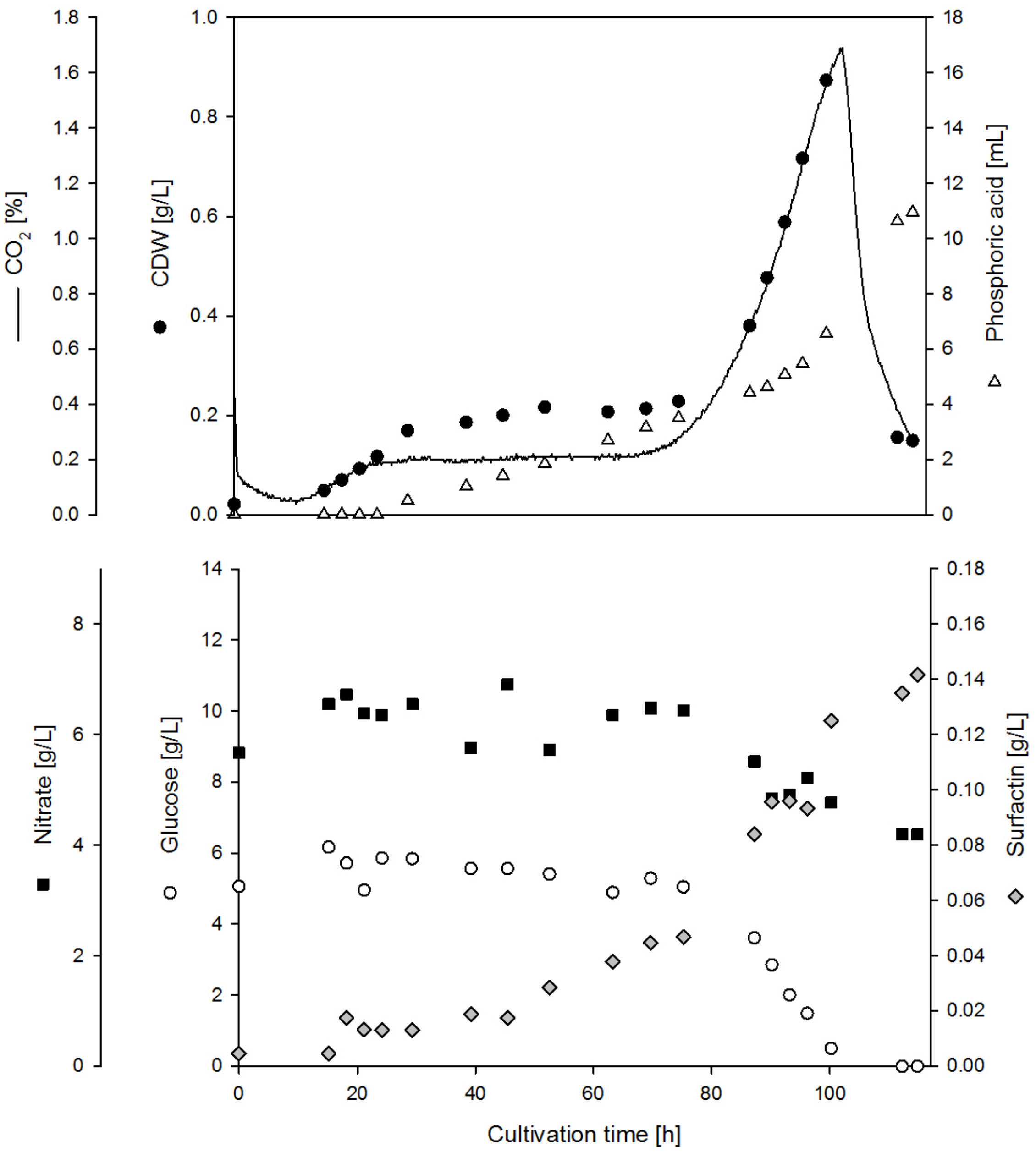

Figure S3 Anaerobic fermentation of *Bacillus subtilis* DSM 10^{T} employing 7.5 g/L glucose Time course of CDW [g/L], CO_2 [%], phosphoric acid [mL], nitrate [g/L] and glucose [g/L] in comparison to produced Surfactin [g/L] during the fermentation process of *Bacillus subtilis* DSM 10^{T} with 7.5 g/L glucose. The values for CDW (black circle), CO_2 (line), phosphoric acid (grey triangle), nitrate (square), glucose (white circle) and Surfactin (grey rhombus) are given as examples of one fermentation

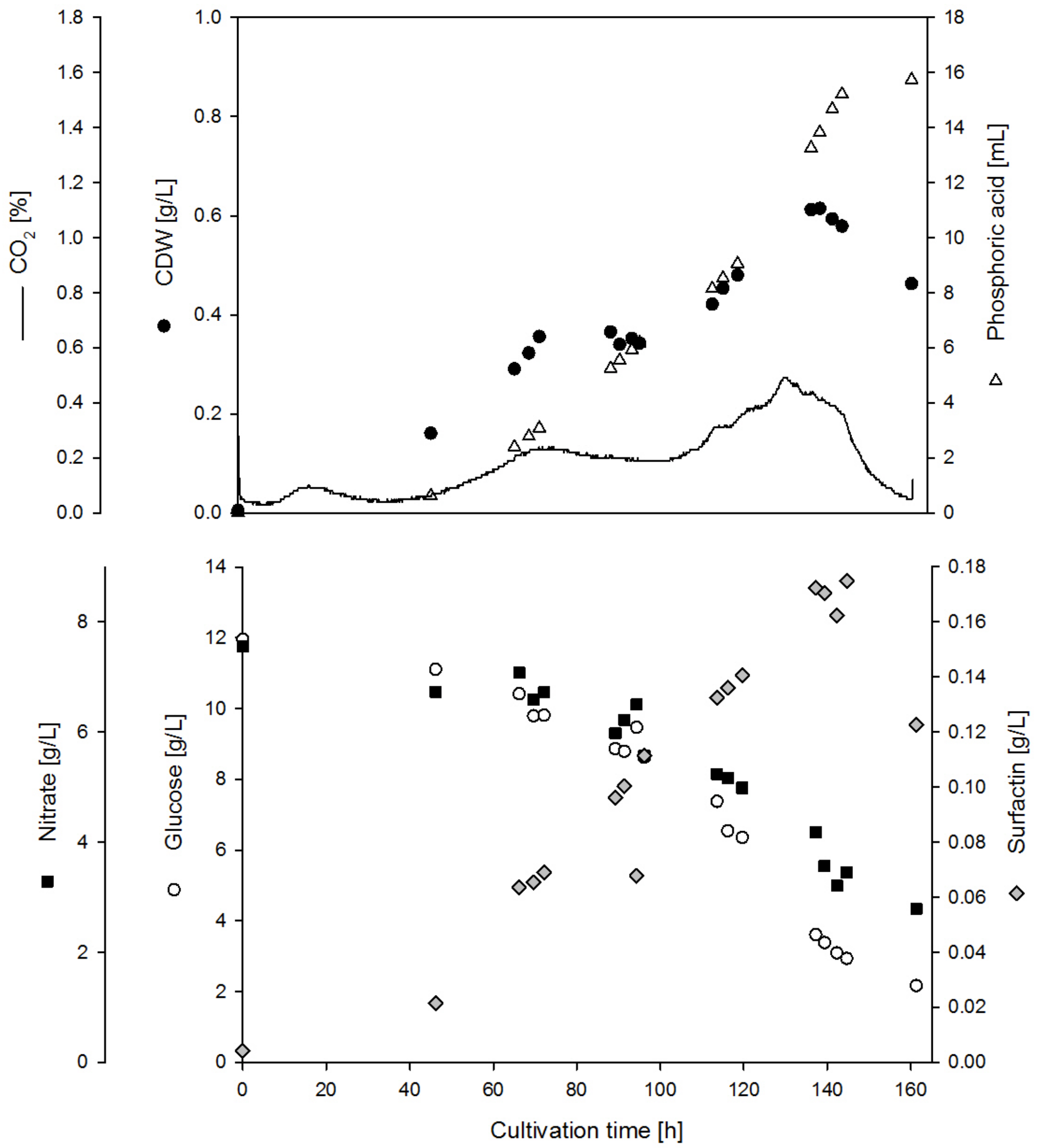

Figure S4 Anaerobic fermentation of *Bacillus subtilis* DSM 10^T employing 10.0 g/L glucose Time course of CDW [g/L], CO_2 [%], phosphoric acid [mL], nitrate [g/L] and glucose [g/L] in comparison to produced Surfactin [g/L] during the fermentation process of *Bacillus subtilis* DSM 10^T with 10.0 g/L glucose. The values for CDW (black circle), CO_2 (line), phosphoric acid (grey triangle), nitrate (square), glucose (white circle) and Surfactin (grey rhombus) are given as examples of one fermentation
